# Electrochemical Sensing of Nitrite Ions Using Modified Electrode by Poly 1,8-Diaminonaphthalene/Functionalized Multi-Walled Carbon Nanotubes

**DOI:** 10.3389/fchem.2022.870393

**Published:** 2022-03-16

**Authors:** Ouissal Salhi, Tarik Ez-zine, Larbi Oularbi, Mama El Rhazi

**Affiliations:** Laboratory of Materials Membranes and Environment, Faculty of Sciences and Technologies, University Hassan II Casablanca, Mohammedia, Morocco

**Keywords:** nitrite detection, conducting polymer, 1,8-Diaminonaphthalene, electrochemical sensor, multi-walled carbon nanotubes

## Abstract

A novel electrochemical sensor based on conducting polymer and multi-walled carbon nanotubes was reported for the detection of nitrite ions (NO_2_^−^). The hybrid material poly 1,8-Diaminonaphthalene (poly 1,8-DAN)/functionalized multi-walled carbon nanotubes (f-MWCNT) was prepared by using a simple electrochemical approach which is based on the deposition of functionalized multi-walled carbon nanotubes (f-MWCNT) on the surface of the electrode followed by the electropolymerization of 1,8-DAN using cyclic voltammetry. The morphology and the electro-catalytic properties of the obtained electrodes were investigated with Fourier Transform Infrared Spectroscopy (FTIR), Transmission Electron Microscopy (TEM), Cyclic Voltammetry (CV), and Electrochemical Impedance Spectroscopy (EIS) showing an improvement of the electronic transfer due to the synergic effect between the proprieties of poly 1,8-DAN and f-MWCNT. Under the optimum experimental conditions, the poly 1,8-DAN/f-MWCNT/CPE exhibited excellent electro-catalytic activity towards nitrite detection. The nitrite anodic peak potential decreased by 210 mV compared to the bare carbon paste electrode. The calibration plot of nitrite detection was linear in the range of concentration from 300 to 6500 nM with a low detection limit of 75 nM.

## Introduction

Nitrite ions; one of the inorganic forms of natural nitrogen; take part of the cycle in which nitrogen takes different forms following several processes such as fixation, assimilation, nitrification, and denitrification (Zhu et al., 2015). It is important to highlight that the industrialization, overpopulation, and the progress of the agri-food sector have led to the accumulation of nitrite in our environment in a very significant way (Berardo et al., 2016). Indeed, it is generated during the decomposition of fertilizers and human waste leading to the contamination of the soil and the groundwater. Nitrite is also used as food preservatives and food coloring under the form of sodium nitrite noted (E250) and constitutes a real threat to the human health. In fact, it has been reported that an excess of this element in the body leads to the conversion of the hemoglobin to methemoglobin, which reduce the amount of oxygen distributed by the blood to the tissues called hypoxia (Katabami et al., 2016). Nitrite ions can also react with amines to form N-nitroamines causing serious health problems such as gastric cancer (Song et al., 2015; Zhang et al., 2019), thyroid cancer (Aschebrook-Kilfoy et al., 2013), and non-Hodgkin's lymphoma (Yu et al., 2020). Moreover, research studies have confirmed a possible relationship between nitrite consumption and neurological damages (Ribeiro et al., 2017). Therefore, there is an urgent need to monitor the presence of nitrite in water and food. For this purpose, the World Health Organization (WHO) has determined a maximum admissible concentration in drinking water of 3 mg/L equivalent to 65.217 µM (Ren et al., 2018). Numerous methods have been developed to detect and quantify nitrite (Wang et al., 2017), such as spectrofluorimetry (Zhan et al., 2019), spectrophotometry (Lo et al., 2017), chemiluminescence (Han and Chen, 2019), chromatography (Lin et al., 2019), capillary electrophoresis (Kalaycıoğlu and Erim, 2016), and electrochemistry (Zhao et al., 2017). The electrochemical techniques possess several advantages such as low cost, rapid response and high sensitivity (Bansod et al., 2017; Oularbi et al., 2019). The electro-oxidation of nitrite leads to the formation of nitrate, unlike its electro-reduction, which can produce several products, and it is limited by the interference of dioxygen (O_2_) and nitrate reduction (NO_3_^−^) (Kesavan et al., 2018). However, the oxidation of nitrite still faces many challenges to overcome, such as the oxidation overpotential, and the slow kinetic of the electronic transfer (Mao et al., 2018). To address this issues, electrochemical sensors and biosensors seem to be a very good alternative. It is, in fact, absolutely necessary to modify the conventional electrodes such as carbon paste (Bijad et al., 2017), screen-printed electrodes (Jaiswal et al., 2017), and glassy carbon electrodes with a suitable agent (Muthumariappan et al., 2017; Velmurugan et al., 2020; Karimi-Maleh et al., 2021). Electrochemical Multi-walled carbon nanotubes (MWCNT), largely used to modify the surface of electrodes for many applications (Mani et al., 2017; Beitollahi et al., 2018; Roy et al., 2018; Rui et al., 2020; Shaikshavali et al., 2020; Baghayeri et al., 2021a); are considered as an ideal choice thanks to their interesting properties. Moreover, they can be combined with metallic nanoparticles, metal organic framework or conducting polymers in order to enhance the sensitivity towards nitrite determination in different matrices (Thirumalraj et al., 2016; Wan et al., 2017; Yu et al., 2019; Baghayeri et al., 2020a; Salagare et al., 2020). Conducting polymers (CPs) constitute a new class of emerging materials and have shown considerable potential for several applications (Gracia and Mecerreyes, 2013; Elbasri and Rhazi, 2015; Salih et al., 2017b; El Rhazi et al., 2018; Salih et al., 2018; Chemchoub et al., 2019; Chemchoub et al., 2020; El Attar et al., 2020; Vagin et al., 2021). They have also shown excellent electrocatalytic activity towards nitrite detection. Recently, several hybrid materials based on CPs have been developed for nitrite sensing (Salhi et al., 2021). Rajalakshmi and John (2015), developed a composite based on 5-amino-1,3,4-thiadiazole-2-thiol and MWCNT for nitrite detection and obtained a very low detection limit of about 0.2 nM. A novel electrochemical approach was also reported in the literature which consists on the combination of poly (3,4-ethylenedioxythiophene) with gold nanoparticles leading to a very good activity toward nitrite oxidation with high stability and selectivity (Lin et al., 2016). Moreover, Shi et al., co-deposited palladium nanoparticles and poly (1.5-Diaminonaphthalene) on MWCNT. The prepared hybrid showed a good electrocatalytic activity towards nitrite oxidation due to its high active surface area and the synergistic effect of Pd nanoparticles, poly 1.5-DAN and MWCNT (Shi et al., 2019). Nevertheless, the main disadvantage of the sensors based on gold and palladium nanoparticles lies in the fact that they are quite expensive.

In this context, we propose a very simple approach which consists on the combination of the two materials (CP and MWCNT) in order to develop an electrochemical sensor; efficient and less expensive; for nitrite determination in water samples. The electropolymerization of 1,8-DAN on carbon nanotubes was carried out using cyclic voltammetry in acidic medium, which allowed us to prepare our working electrodes in less than 1 hour. The morphology and the electrochemical behavior of the modified electrodes were investigated using various techniques such as Fourier transform infrared spectroscopy (FTIR), transmission electron microscopy (TEM), cyclic voltammetry (CV), and electrochemical impedance spectroscopy (EIS). The electrocatalytic activity of poly 1,8-DAN/f-MWCNT/CPE towards nitrite oxidation was examined using both cyclic voltammetry and amperometry.

## Experimental

### Chemical and Reagents

Graphite powder, paraffin oil, 1,8-Diaminonaphthalene (1,8-DAN), potassium Ferri/Ferrocyanide (K3Fe(CN)_6_/K_4_Fe(CN)_6_.3H_2_O; ACS reagent >99%), disodium hydrogen phosphate heptahydrate (Na2HPO4.7H2O) and sodium dihydrogen phosphate dihydrate (NaH_2_PO_4_.2H_2_O) were obtained from Sigma Aldrich. Potassium chloride (KCl), hydrochloric acid (HCl, 37%), nitric acid (HNO_3_, 69%), and sulfuric acid (H_2_SO_4_, 98%) were procured from Labo Chimie. Sodium nitrite (NaNO_2_, ACS reagent >98%) was acquired from Panreac Quimica. Bi-distillated water was used to prepare all the solutions including 0.1 M of phosphate buffer solution (PBS, pH 7.2), which was used as the electrolyte.

### Functionalization of Multi Walled Carbon Nanotubes

In order to functionalize multi-walled carbon nanotubes, an appropriate amount of MWCNT was dispersed in a solution of concentred H_2_SO_4_ and HNO_3_, with a volume ratio of 1:3. The suspension was ultrasonicated for a few minutes, then left under magnetic stirring over-night. Afterwards, the functionalized multi walled carbon nanotubes (f-MWCNT) were filtered using a Millipore polycarbonate membrane with a diameter of 0.22 µm, and washed with bi-distillated water until a neutral pH of the filtrate was obtained. Eventually, the f-MWCNT were dried at the oven at 50°C for 5 h.

### Preparation of the Modified Electrodes

Firstly, the carbon paste electrode (CPE) was prepared by mixing 1 g of graphite powder with 30% paraffin oil w/w in a mortar until the formation of a homogeneous paste (Salih et al., 2017a; Oularbi et al., 2020; El Attar et al., 2021). Subsequently, the paste was placed in a 3 mm diameter Teflon tube. Then, it was polished until the obtention of a smooth surface. The aqueous suspension of f-MWCNT was prepared by dispersing 1 mg of f-MWCNT in 1 ml of distillated water. Afterward, 20 µL of the suspension was dropped onto the surface of CPE, then dried at the oven at 50°C.

The electrochemical deposition of poly (1,8-DAN) was carried out in a 0.1 M HCl solution containing 5 mM of the monomer (1,8-DAN) using cyclic voltammetry (CV) by sweeping the potential from −0.5 to 1 V vs. Ag/AgCl for 15 cycles at a scan rate of 50 mV/s.

### Electrochemical Measurements and Physicochemical Characterization

All the electrochemical measurements including cyclic voltammetry (CV), electrochemical impedance spectroscopy (EIS), and amperometry were performed using a PalmSens4 connected to a PSTrace 5.3 software. A traditional three electrodes system was used, which consists of a modified carbon paste electrode (CPE) as the working electrode, a platinum disk as the counter electrode, and a silver/silver chloride (Ag/AgCl) as the reference electrode.

The CV measurements were carried out in a 0.5 M KCl solution containing 10 mM [Fe(CN)_6_]^3−/4−^ at an applied potential ranging from −0.4 to 0.8 V vs. Ag/AgCl, and in 0.1 M phosphate buffer (PBS, pH 7.2) containing 0.1 M KCl and 1 mM NO_2_^−^, sweeping the potential from −0.2 to 1.1 V vs. Ag/AgCl at different scan rates. Electrochemical impedance spectroscopy (EIS) measurements were performed in 0.1 M PBS (pH 7.2) containing 0.1 M KCl and 1 mM NO_2_^−^ at a fixed potential of 0.85 V vs. Ag/AgCl, a frequency range from 10 mHz to 63 kHz and an amplitude of 10 mV. The amperometry measurements were conducted in 0.1 M PBS containing 0.1 M KCl at a fixed potential of 0.9 V vs. Ag/AgCl under stirring. It should be noted that all the experiments were performed at room temperature.

Fourier Transform Infrared spectroscopy (FTIR) using an Affinity-1S SHIMADZU spectrometer equipped with a golden gate single reflection attenuated total reflectance (ATR) accessory. FTIR spectra were recorded in the range of 500–4000 cm⁻^1^ at a resolution of 16 cm⁻^1^ and were used to determine the structural properties of f-MWCNT and poly 1,8-DAN/f-MWCNT. The morphological properties of the electrocatalysts were characterized using Transmission Electron Microscopy-EDX (TALOS F200S).

## Results and Discussions

### Polymerization of 1,8-DAN

**FIGURE 1:**
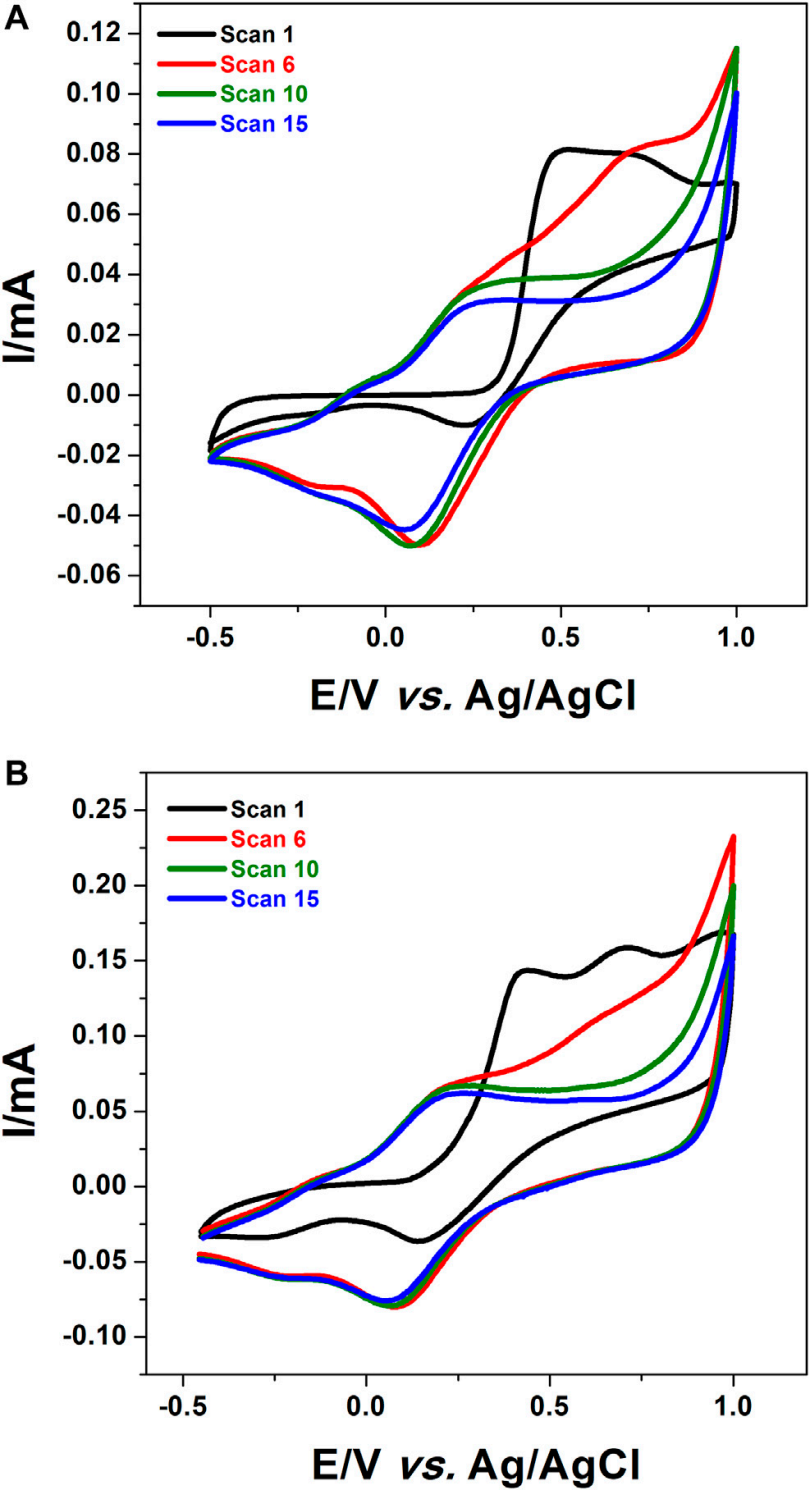
CV of **(A)** CPE and **(B)** f-MWCNT/CPE in 0.1 M HCl containing 5 mM 1,8-DAN for 15 cycles at a scan rate of 50 mV/s.

In order to polymerize 1,8-Diaminonaphthalene onto the surface of CPE and f-MWCNT/CPE, cyclic voltammetry technique was performed in a 0.1 M HCl solution containing 5 mM of the monomer (1,8-DAN) by sweeping the potential from −0.5 to 1 V vs. Ag/AgCl at a scan rate of 50 mV/s for 15 scans. The formation of a brown film on each electrode was observed indicating the formation of poly 1,8-DAN (Oyama et al., 1989). The cyclic voltammograms obtained at CPE and f-MWCNT/CPE were illustrated in Figure 1. The bare electrode (Figure 1A) shows a peak current at +0.5 V vs. Ag/AgCl corresponding to the irreversible electrooxidation of 1,8-DAN which disappears completely after the sixth scan giving rise to new oxidation-reduction peaks which appear at about +0.05 and +0.2 V vs. Ag/AgCl. This is consistent with the previous results found by Majid et al. (2003). However, at the f-MWCNT/CPE as it is shown in Figure 1B, during the first scan, a peak current was observed at + 0.4 V vs. Ag/AgCl which corresponds to the irreversible oxidation of the monomer. The shift of about 100 mV observed on CPE modified by f-MWCNT can be attributed to the 3-dimensional network of f-MWCNT which increases the electrode surface area. It should be noted that a second peak was observed at 0.7 which may correspond to the second oxidation of the amine group. These results are in agreement with the work of Lee et al. (1992), when using platinum electrode for the polymerization of 1,8-DAN. It is important to highlight that after the modification of the electrode with f-MWCNT, the current intensities increased by about 76%. This may be attributed to the higher and good electrical conductivity of f-MWCNT and/or to the 3-dimensional network and large surface area of the f-MWCNT (Guo et al., 2020).

**FIGURE 2:**
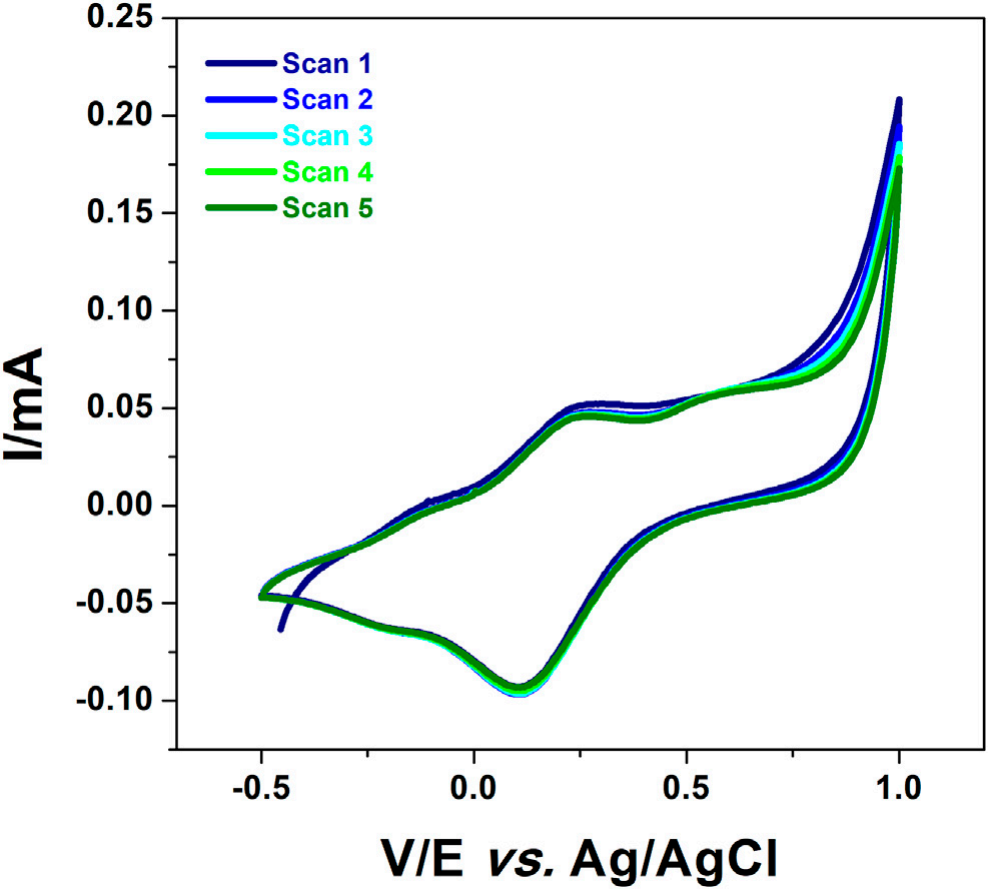
CV of poly 1,8-DAN/f-MWCNT/CPE in 0.1 M HCl at a scan rate of 50 mV/s.

In order to check the formation of a stable polymer, the prepared electrode was then placed in a 0.1 M HCl solution free monomer and CV was performed under the potential range of −0.5–1 V at a scan rate of 50 mV/s for 5 cycles. Figure 2 shows the results obtained on poly 1,8-DAN/f-MWCNT/CPE. It can be seen that the voltammograms kept the same shape after consecutive scans revealing the oxidation/reduction peaks of poly 1,8-DAN in acidic medium, which indicates that a stable film of poly 1,8-Diaminonapththalene was synthesized on f-MWCNT/CPE (Salih et al., 2017b).

### FTIR and TEM Characterization of Poly 1,8-DAN/f-MWCNT

**FIGURE 3:**
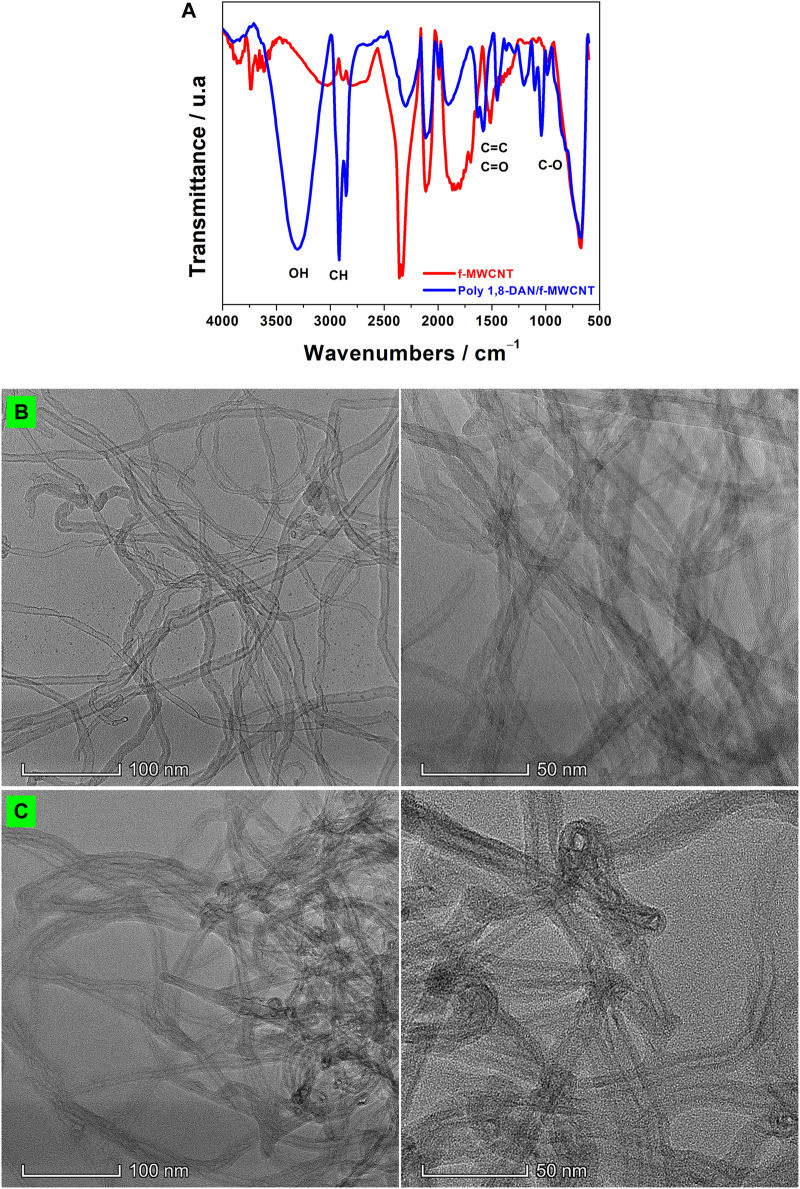
**(A)** FTIR spectra of f-MWCNT (red line), and poly 1,8-DAN/f-MWCNT (blue line). TEM images of **(B)** f-MWCNT, and **(C)** poly 1,8-DAN/f-MWCNT.

FTIR spectroscopy was used to confirm the functionalization of MWCNT with carboxylic groups, and also to confirm the deposition of poly 1,8-DAN film (Figure 3A). The FTIR of f-MWCNT (red line) showed two absorption bands at 2334 and 2361 cm^−1^, which correspond to the hydrogen of the carboxylic group (-COOH). In addition to this, two other bands were noticed at 976 and 1741 cm^−1^, which are attributed respectively to the C=O and C-O of the -COOH. Another band was observed at 3600 cm^−1^ indicating the presence of the hydroxylic group -OH of -COOH (Oularbi et al., 2017; Oularbi, 2018; El Attar et al., 2022). These results demonstrated that the MWCNT were successively functionalized. The structural properties of poly 1,8-DAN/f-MWCNT have also been investigated using FTIR spectroscopy (blue line). It can be seen that new peaks appeared after the electrodeposition of the polymer. In fact, the absorption band located in 3309 cm^−1^ corresponds to the N-H stretching. Whereas, the peaks that appeared at 1490 and 1581 cm^−1^ are attributed to the C-C of the polymer. Furthermore, the band of 1203 indicates the C-N stretching (Pałys et al., 1997). These results confirm the formation of poly 1,8-DAN on the surface of f-MWCNT/CPE. The morphologies of f-MWCNT/CPE and poly 1,8-DAN/f-MWCNT/CPE were examined using TEM (Figures 3B,C). The corresponding images (Figure 3B) reveal a slight increase of the carbon nanotubes diameter after the electrochemical deposition of poly 1,8-DAN.

The thickness of the polymeric film (d) was also determined using Eq. 1 described as follow (Halim et al., 2019):

$$\text{d}=\left(\text{Q}\times {\text{M}}_{\text{w}}\right)/\left(\text{n}\times \text{F}\times \text{A}\times \varphi \right)$$ (1)

Where M_w_ is the molar mass of the monomer (158.2 g/mol), Q is the electrical charge associated to the polymer formation, n is the number of the involved electrons (2) (Lee et al., 1992), F is the Faraday (96485 Q/mol), A is the surface area of the working electrode, and φ is the monomer density (1.12 g/cm^3^). The thickness of poly 1,8-DAN film deposited on the surface of f-MWCNT/CPE was found to be 22 nm, which indicates the formation of a thin film on the surface. This thickness is in agreement with the results found using the transmission electron microscopy.

### Electrochemical Characterization of Poly 1,8-DAN/f-MWCNT

#### Cyclic Voltammetry Characterization in [Fe(CN)_6_]^3-/4-^

**FIGURE 4:**
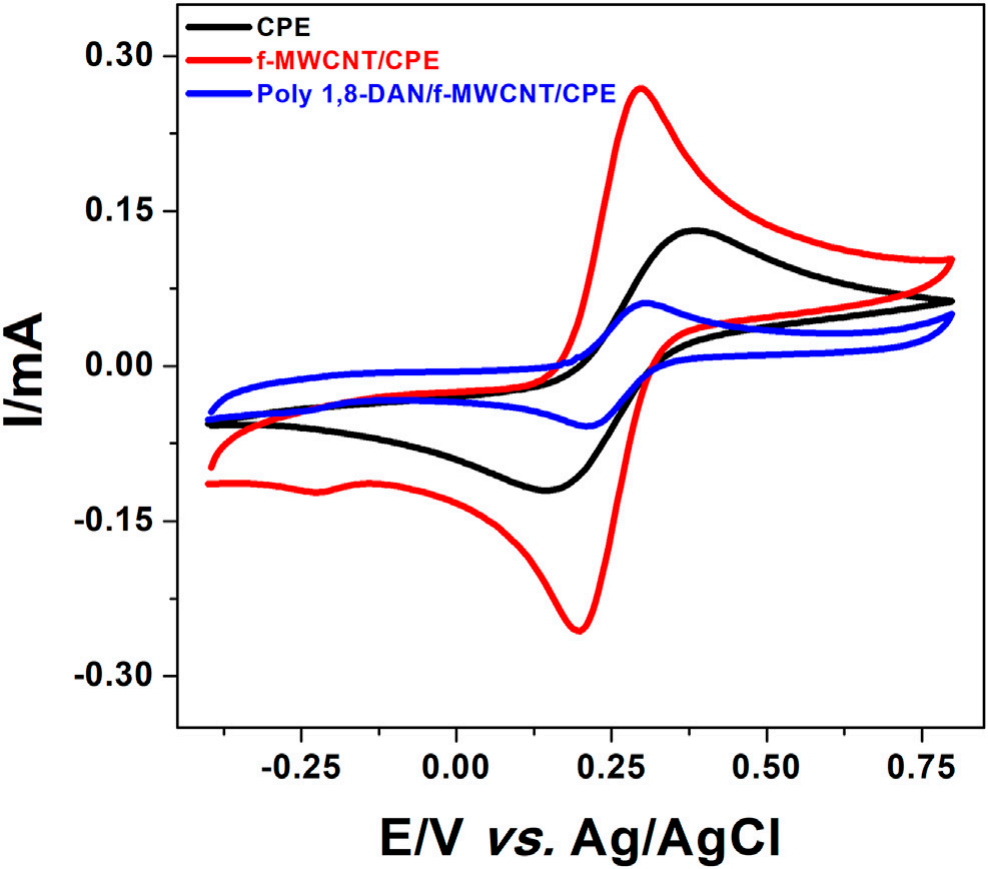
CV of CPE (black line), f-MWCNT/CPE (red line), and poly 1,8-DAN/f-MWCNT/CPE (blue line) in 0.5 M of KCl containing 10 mM of [Fe(CN)_6_]^3−/4−^ at a scan rate of 50 mV/s.

The preliminary investigations were aimed towards comparing the electrochemical responses of ferri-ferrocyanide system on different electrodes. In this perspective, CV measurements were performed in 0.5 M KCl containing 10 mM [Fe(CN)_6_]^3−/4−^ at CPE, f-MWCNT/CPE, poly 1,8-DAN/f-MWCNT/CPE in the potential range from −0.4–0.8 V vs. Ag/AgCl. As can be seen in Figure 4 and Table 1, the peak-to-peak separation on CPE (black line) is equal to 223 mV, which indicate a slow electronic transfer. On the f-MWCNT/CPE, the peak-to-peak separation was reduced to reach the value of 98 mV (red line) and the current intensities increased by about 40%. This behavior could be explained by the large specific surface area and high electrical conductivity offered by f-MWCNT. The same behavior was observed by Oularbi et al. (2017), when using Polypyrrole and Carbon nanofibers. A surprising fact was observed after the electrodeposition of poly 1,8-DAN on f-MWCNT/CPE (blue line). A dramatic decrease of the value of ΔE_p_ was found, around 91 mV, which is about more than 3 times smaller than the bare CPE. The only possible explanation is that the decrease in ΔE_p_ is probably due to the improvement of the electronic transfer at the interface electrode-solution due to the presence of a large amount of amine groups all along the polymer backbone (Wang et al., 2016). Indeed, It has been proved that an amine-rich surface enhances the electronic transfer (Li et al., 2017; Ning et al., 2019). Contrary to what one might expect to find, a low current compared to CPE and f-MWCNT/CPE was obtained due to the low conductivity of poly 1,8-DAN at neutral pH (Majid et al., 2003).

**TABLE 1:** Comparison between I_pa_, I_pc_ and the peak-to-peak separation (Δ_Ep_) for CPE, f-MWCNT/CPE and 1,8-DAN/f-MWCNT/CPE.

Electrode	I_pa_/µA	I_pc_/µA	Δ_Ep_/V
Bare CPE	235	196	0.332
f-MWCNT/CPE	393	387	0.098
Poly 1,8-DAN/f-MWCNT/CPE	40	47	0.091

**FIGURE 5:**
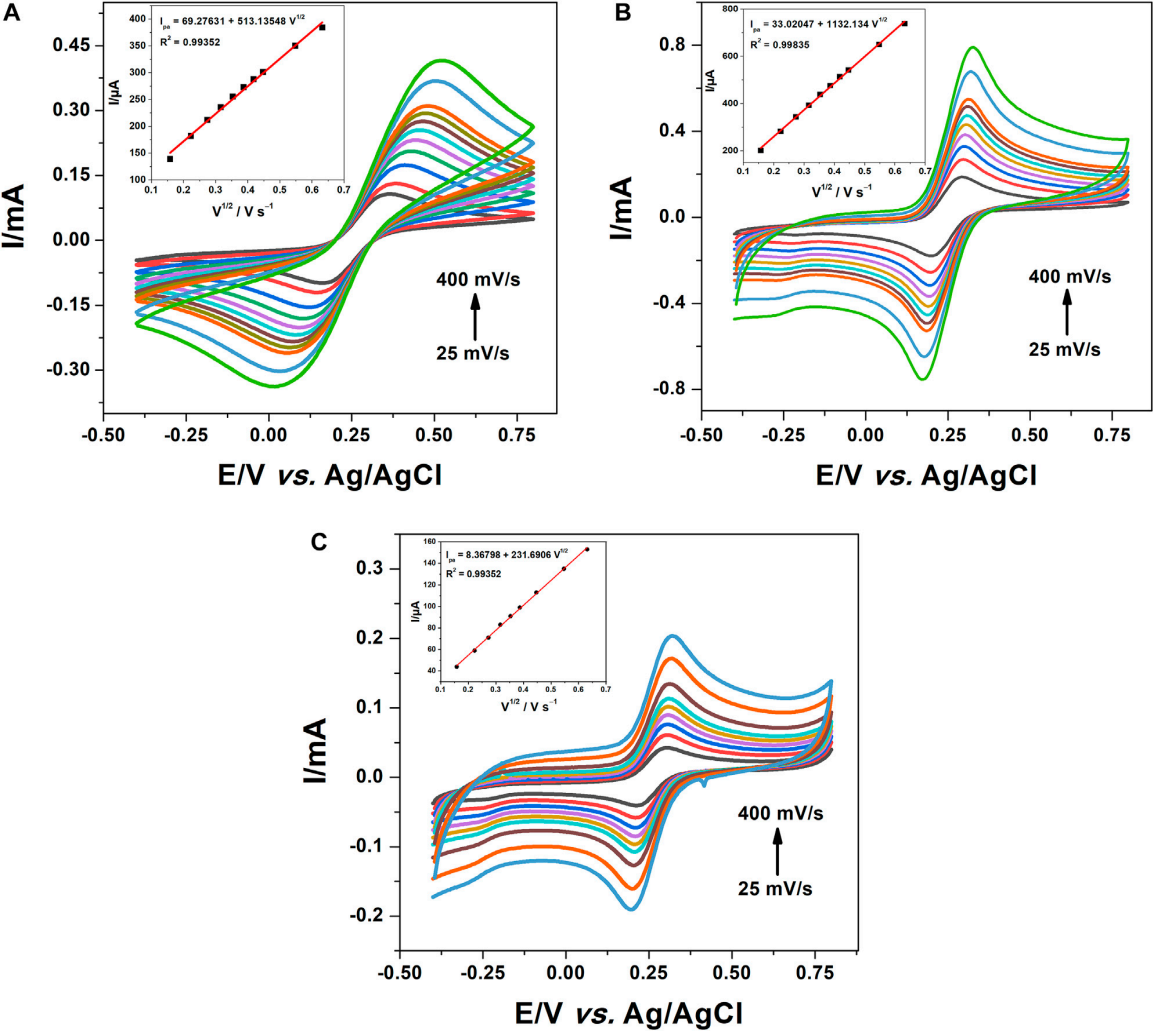
CV of **(A)** CPE, **(B)** f-MWCNT/CPE, and **(C)** poly 1,8-DAN/f-MWCNT/CPE in 0.5 M of KCl containing 10 mM of [Fe(CN)_6_] ^3−/4−^ at different scan rates.

The specific surface area of all the electrodes was calculated using the Randles and Sevcik Eq. 2 (Analytical Electrochemistry, Wang, 2006) as shown in Figure 5:

$${\text{I}}_{\text{p}}=2.69\times 10^{5}\times {\text{n}}^{3/2}\times \text{A}\times \text{C}\times {\text{D}}^{1/2}\times {\text{V}}^{1/2}$$ (2)

Where, the number of electrons transferred (n) is equal to 1, the concentration (C) is equal to 10 mM and the diffusion coefficient (D) is equal to 6.7 10^–6^ cm^2^/s (Halim et al., 2019). The specific surface areas were found to be 0.073, 0.16^2^, and 0.033 cm^2^, respectively for CPE, f-MWCNT/CPE, and poly 1,8-DAN/f-MWCNT/CPE. Indeed, the specific surface of f-MWCNT/CPE was about two times higher than the bare CPE. This was due to the large specific surface of multi-walled carbon nanotubes (Shi et al., 2019). It is important to mention that the electrode modified with poly 1,8-DAN and f-MWCNT has the smallest surface area. This phenomena can be explained by the low electrical conductivity of polymer, and the lack of active sites of the poly (1,8-DAN) at neutral pH (Majid et al., 2003).

### Electrooxidation Behavior of Nitrite on the Poly 1,8-DAN/f-MWCNT

**FIGURE 6:**
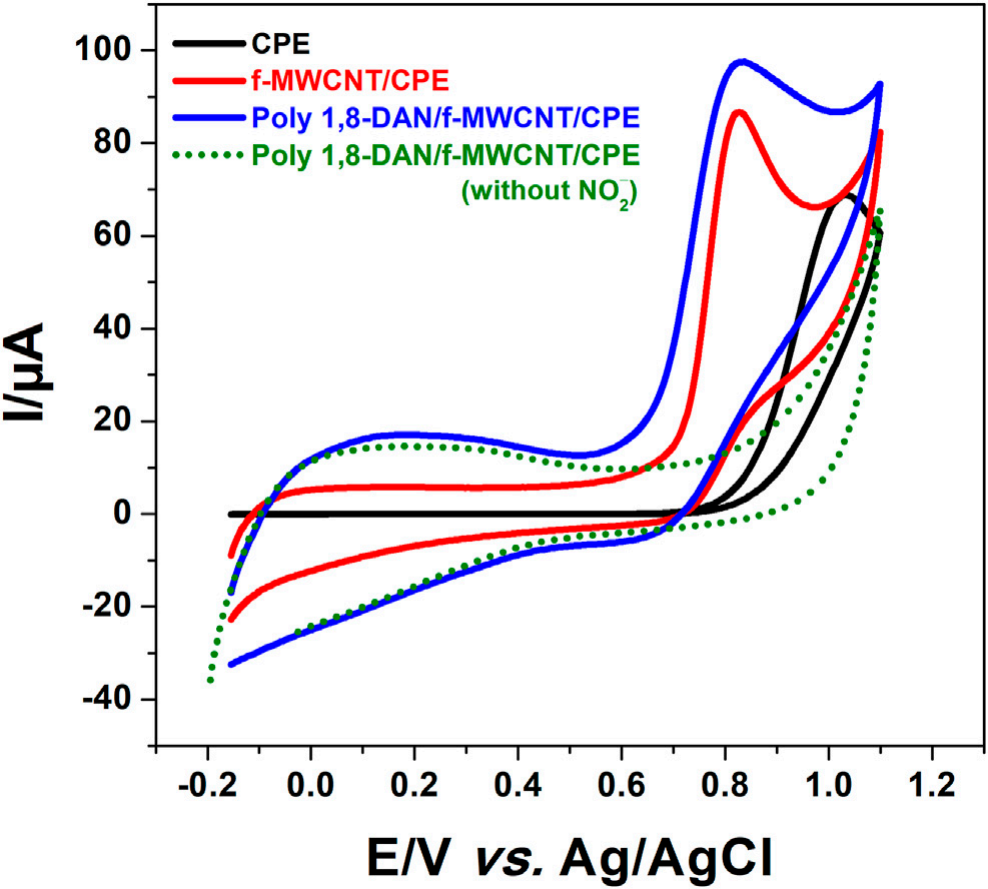
CV of CPE (balck line), f-MWCNT/CPE (red line), and poly 1,8-DAN/f-MWCNT/CPE in 0.1 M PBS containing 0.1 M KCl (green dots) and 1 mM NO_2_^−^ (blue line) at pH 7, 2 and a scanning speed of 50 mV/s.

**FIGURE 7:**
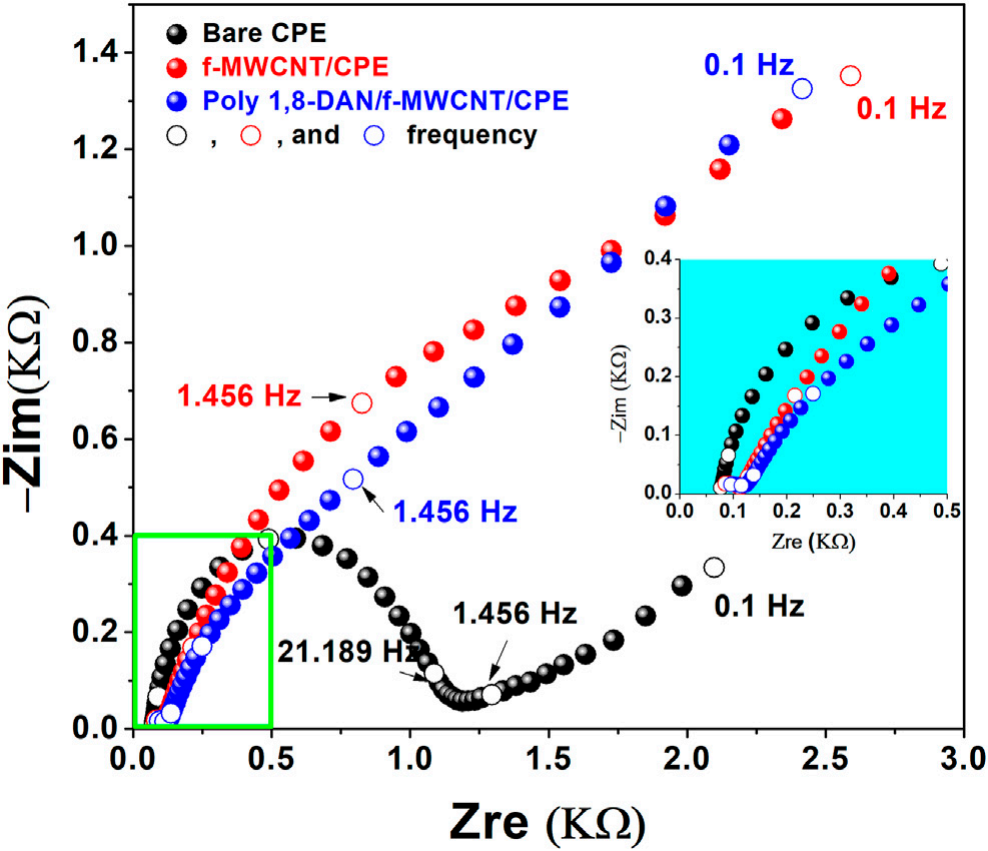
Nyquist plots of CPE (black line), f-MWCNT/CPE (red line), and poly 1,8-DAN/f-MWCNT/CPE (blue line) in 0.1 M PBS containing 0.1 M KCl and 1 mM NO_2_^−^ at pH 7.2 and a fixed Potential of 0.85 V vs. Ag/AgCl.

After the characterization of our electrodes, the behavior of the different electrodes in presence of nitrite was investigated using CV in a pH 7.2, 0.1 M phosphate buffer solution containing 0.1 M KCl and 1 mM nitrite. Figure 6 presents the corresponding voltammograms at the bare CPE, f-MWCNT/CPE, and poly 1,8-DAN/f-MWCNT/CPE. An irreversible peak at 1.04 V vs. Ag/AgCl (black line) was obtained at bare carbon paste electrode which corresponds to the irreversible oxidation of nitrite. As expected, the peak potential was shifted to more negative value (210 mV) after the modification of CPE with f-MWCNT (red line) with an important increase in the current intensity. After the electrodeposition of poly 1,8-DAN on the surface of f-MWCNT/CPE (blue line). The same results were observed concerning the peak potential with a further increase of the current intensity, indicating that nitrite is much easier to oxidase on the hybrid material. Similar results were obtained by Huang et al., using gold nanoparticles combined with poly (3-methylthiophene), which was explained by the synergistic effect of combining both gold nanoparticles and conducting polymers. It seems that the carbon nanomaterials combined with conducting polymers enhance the electronic transfer as observed by many authors (Zheng et al., 2009; Rajalakshmi and John, 2015, 2; Arulraj et al., 2018). To better understand this phenomenon, electrochemical impedance spectroscopy (EIS) was conducted in the same solution at an applied potential of 0.85 V vs. Ag/AgCl with a small amplitude of 10 mV. Figure 7 presents the corresponding Nyquist plots. Indeed, it can be seen that the charge transfer resistances (R_ct_) decreased from 3277 Ω to 11 Ω after the modification of CPE with poly 1,8-DAN/f-MWCNT, which is 300 times smaller. These results are in good agreement with the CV measurements and suggests that the prepared hybrid material promotes the electronic transfer between the supporting electrolyte and the electrode.

### Electrooxidation Mechanism of Nitrite on the Poly 1,8-DAN/f-MWCNT

**FIGURE 8:**
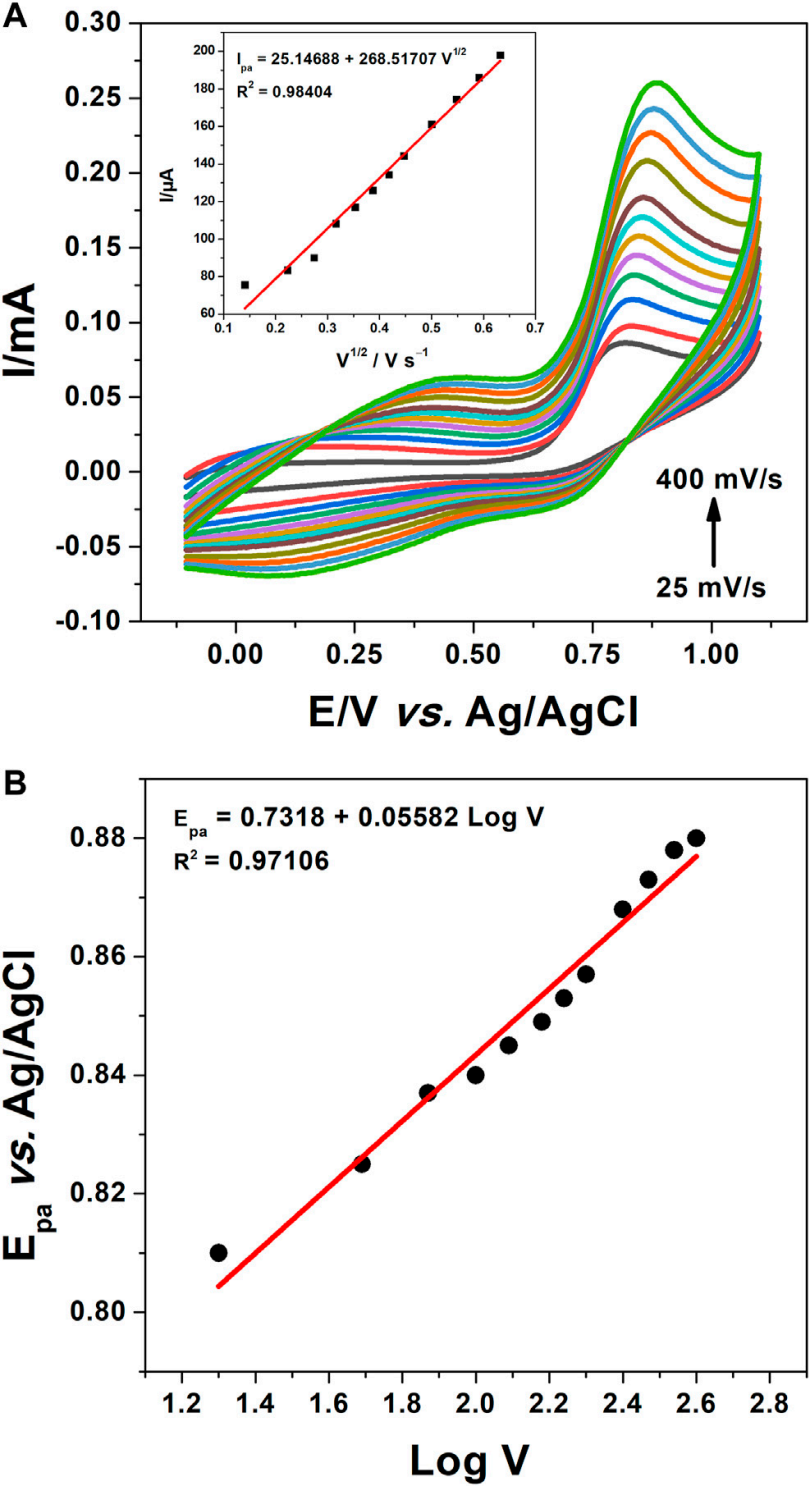
**(A)** CV of poly 1,8-DAN/f-MWCNT/CPE in 0.1 M PBS containing 0.1 M KCl and 1 mM NO_2_^−^ at pH 7.2 and scanning rates from 25 to 400 mV/s with the plot of nitrite oxidation peak current versus V^1/2^, **(B)** the plot of the peak current versus Log(V).

In order to establish the reaction mechanism of nitrite oxidation, cyclic voltammetry experiments were recorded on poly 1,8-DAN/f-MWCNT/CPE. The corresponding graphs shown in Figure 8A present the variation of the oxidation current with the square root of the scan rate.

It can be seen that the nitrite oxidation peak increased linearly with the square root of the scan rate according to the equation I_pa_ = 25.14688 + 268.51707 V^1/2^ with a correlation coefficient of 0.98404 which suggests that the electrochemical reaction is controlled by a diffusion process (Baghayeri et al., 2020b; Baghayeri et al., 2021b). It should be noted that a shift in the oxidation peak potential (E_pa_) to more positive values with the scan rates confirms the irreversibility of the nitrite oxidation reaction as mentioned by others authors (Zhao et al., 2017; Shi et al., 2019). The number of the transferred electrons can be calculated by plotting the E_pa_ *vs.* Log V. E_pa_ varied linearly with the logarithm of the scan rate as described by the equation E_pa_ = 0.7318 + 0.05582 Log V with a correlation coefficient of 0.97106 (Figure 8B). The number of the transferred electrons during the oxidation reaction of nitrite was then calculated according to Laviron’s Eq. 3 (Analytical Electrochemistry, Wang, 2006):

$${\text{E}}_{\text{pa}}{=\text{E}}^{\text{°}}+\left(\text{RT}/\text{anF}\right)\text{ln}\left({\text{RTK}}^{\text{°}}/\text{αnF}\right)+2\text{.}3\left(\text{RT}/\text{αnF}\right)\text{log}\left(\text{v}\right)$$ (3)

Where n is the number of the transferred electrons, α is the electron transfer coefficient which is taken as 0.5 in a totally irreversible reaction (Shi et al., 2019), *v* is the scan rate, and E°’ is the formal potential. Whereas R, T and F have their conventional meaning. The number of electron n involved in the oxidation reaction of nitrite was calculated to be about 2 (2.08), which demonstrates that the electrooxidation of nitrite requires the transfer of two electrons according to the following reactions noted (4) and (5). These results are in agreement with the literature (Arulraj et al., 2018):

$${2\text{NO}}_{2}^{-}↔{2\text{NO}}_{2}{+2\text{e}}^{-}$$ (4)

$${2\text{NO}}_{2}{+\text{H}}_{2}\text{O}\to {\text{NO}}_{3}^{-}{+\text{NO}}_{2}^{-}{+2\text{H}}^{+}$$ (5)

### Calibration Curve and Real Sample Analysis

**FIGURE 9:**
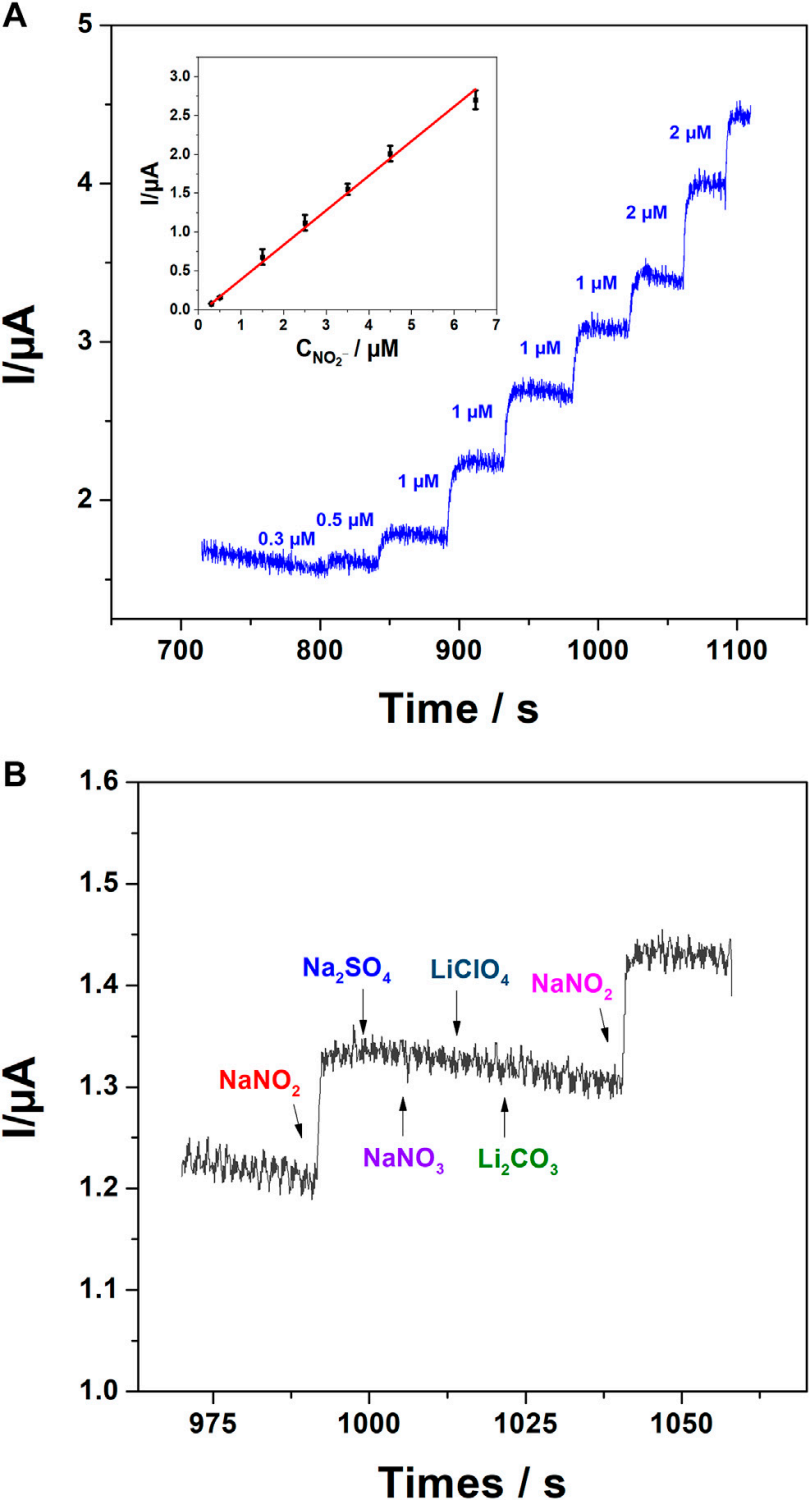
**(A)** Amperometry of poly 1,8-DAN/f-MWCNT/CPE in 0.1 M PBS containing 0.1 M KCl at pH 7.2 with a fixed potential of 0.9 V vs. Ag/AgCl at consecutive additions of nitrite, insert the calibration plot of the current versus nitrite concentrations. **(B)** Effect of some interference components on nitrite response.

After the characterization of our sensor, and in order to evaluate the advantages offered by f-MWCNT and poly 1,8-DAN, amperometry was chosen as a sensitive technique to detect nitrite in aqueous solution (Liu et al., 2011; Rajalakshmi and John, 2015; Zhang et al., 2015; Fan et al., 2017). Indeed, amperometry was performed in 0.1 M PBS pH 7.2 containing 0.1 M KCl at a fixed potential of 0.9 V vs. Ag/AgCl under continuous stirring. The corresponding graph shown in Figure 9A illustrates the variation of the current intensities versus time after successive additions of nitrite. The oxidation current increased linearly with nitrite concentration in a range between 300 and 6500 nM according to equation I = −0.0537 + 0.44453 [NO_2_⁻] with a correlation coefficient of 0.99722, a relative standard deviation (RSD) of 2.36% (*n* = 3 tests), and a sensitivity of 0.44453 μA/μM. The detection limit was calculated by the equation LOD = 3 × SD/P. where SD is the standard deviation, and P the slope of the calibration curve, and it was found to be 75 nM.

Prior to the application in real samples, the selectivity of the developed sensor was investigated in the presence of some components such as NaNO_3_, Na_2_SO_4_, LiClO_4_, and Li_2_CO_3_. From the obtained results (Figure 9B), we can assume that these components did not interfere with the nitrite response. Subsequently, the effectiveness application of the developed hybrid sensor for detection of nitrite in real samples was tested in tap water. The determination of nitrite was based on the method of dosed additions, and the concentration of nitrite was found to be 0.28667 ± 0.01528 µM with an RSD of about 5.3% (3 tests) which suggests that our sensor can be used directly in real samples, and without any pretreatment of water. The detection limit, linearity range, and the detection technique of different hybrids reported in the literature are summarized in Table 2. Our material seems to be a good choice for nitrite determination in water samples.

**TABLE 2:** Comparison of the electrocatalytic performance of different hybrid materials.

Catalyst	Technique	Linear range/µM	Detection limit/µM	Refs.
p-ATT/f-MWCNT	Amperometry	0.001–1.0	0.0002	Rajalakshmi and John (2015)
AuNPs/PEDOT	Amperometry	0.2–1400.0	0.06	Lin et al. (2016)
PdNPs-poly (1.5-DAN)/MWCNT	Amperometry	0.25–100	0.08	Shi et al. (2019)
rGO/AuNPs	Chronoamperometry	1.0–6000.0	0.13	Jian et al. (2018)
Pd/Fe3O4/Poly DOPA/rGO	Amperometry	2.5–6470.0	0.5	Zhao et al. (2017)
AuNPs/MWCPE	Amperometry	0.05–250	0.01	Afkhami et al. (2014)
Au-rGO/PDDA	DPV	0.05–8.5	0.04	Jiao et al. (2015)
Poly (1,8-DAN)/f-MWCNT	Amperometry	0.3–6.5	0.075	This Work

## Conclusion

In summary, a low-cost sensor based on 1,8-Diaminonaphthalene electrodeposited on f-MWCNT/CPE was developed for a rapid and effective determination of nitrite in aqueous solutions. The characterization of the electrode was performed using different techniques such as FTIR, TEM, CV and EIS. The FTIR and TEM characterizations confirm the presence of the polymer on the surface of our sensor, while the electrochemical measurements revealed that the combination of f-MWCNT and poly 1,8-DAN improves the electronic transfer, and has very good electro-catalytic activity towards nitrite oxidation. The sensor was then applied for detection of nitrite showing a good linearity, selectivity and sensitivity with a very low detection limit of 75 nM. It should be highlighted that the sensor can be used directly and without any pre-treatment of water samples. More studies are undergoing to enhance the linearity range and to extend the applicability of this sensor.

## Data Availability

The original contributions presented in the study are included in the article/Supplementary Material, further inquiries can be directed to the corresponding author.
